# Supplementary figures and images for: TerrestrialMetagenomeDB: a public repository of curated and standardized metadata for terrestrial metagenomes
Source: Nucleic Acids Res. 2019 Nov 15;48(D1):D626–32. doi: 10.1093/nar/gkz994 (PMC7145636; doi:10.1093/nar/gkz994)

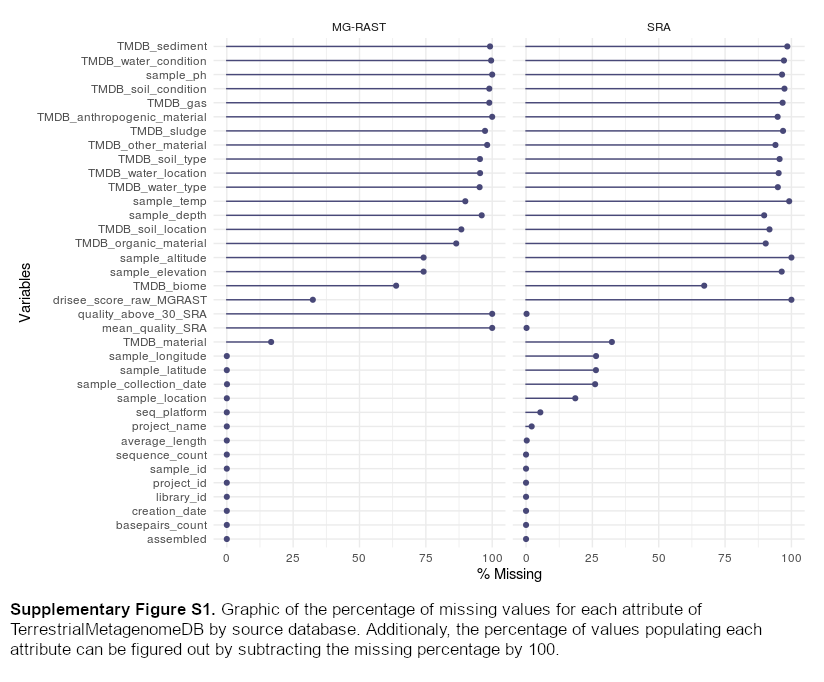

Supplement: gkz994_Supplemental_Files [file gkz994_supplemental_files.zip › Suppl_Figure_S1.png]
